# Increased risk of suicide in New South Wales men with prostate cancer: Analysis of linked population-wide data

**DOI:** 10.1371/journal.pone.0198679

**Published:** 2018-06-13

**Authors:** David P. Smith, Ross Calopedos, Albert Bang, Xue Qin Yu, Sam Egger, Suzanne Chambers, Dianne L. O’Connell

**Affiliations:** 1 Cancer Research Division, Cancer Council NSW, Sydney, New South Wales, Australia; 2 Sydney School of Public Health, University of Sydney, Sydney, New South Wales, Australia; 3 Menzies Health Institute Queensland, Griffith University, Gold Coast, Queensland, Australia; 4 Wollongong Hospital, Wollongong, New South Wales, Australia; 5 School of Medicine and Public Health, University of Newcastle, Newcastle, New South Wales, Australia; Queensland University of Technology, AUSTRALIA

## Abstract

**Background:**

An elevated risk of suicide after a diagnosis of prostate cancer has been reported previously in the USA and Sweden. We aimed to identify whether prostate cancer survivors resident in New South Wales Australia are at higher risk of suicide and if so, who is most at risk.

**Methods:**

Data were obtained from the New South Wales (NSW) Cancer Registry for all men diagnosed with prostate cancer in NSW during 1997 to 2007. These were linked by the Centre for Health Record Linkage (CHeReL) to Australian Bureau of Statistics Mortality Data to the end of 2007 to determine vital status and cause of death. We compared the number of suicides observed for prostate cancer survivors with the expected number of suicides based on age- and calendar year- specific rates for the NSW male population using standardised mortality ratios (SMRs). Suicide rate ratios (RR) by disease and patients’ characteristics were estimated using multivariable negative binomial regression to determine the most at risk groups.

**Results:**

During the study period 51,924 NSW men were diagnosed with prostate cancer. Forty nine of these men were subsequently recorded as committing suicide up to 10 years after diagnosis with an SMR of 1.70 (95% CI:1.26–2.25). Twenty six (53%) of these suicides occurred within 12 months after diagnosis. Risk diminished over time since diagnosis (RR in 1–2 years after diagnosis = 0.29, 95% CI: 0.12–0.71, 2–4 years RR = 0.30, 95% CI: 0.14–0.16 and 4+ years RR = 0.26, 95% CI: 0.11–0.60 compared with <1 year since diagnosis). Men with non-localised disease had a higher risk of suicide compared to men with localised disease (RR = 2.68, 95% CI: 1.15–6.23). Men living outside major cities had lower risk of suicide compared to those resident in major cities (rate ratio = 0.42, 95% CI: 0.20–0.87). Single, divorced, widowed or separated men were more likely to commit suicide than married men (RR = 4.18, 95% CI: 2.36–7.42).

**Conclusion:**

Risk of suicide is higher for NSW men diagnosed with prostate cancer than the general age matched male population. Vulnerable or lonely men and those with pre-existing depression or suicidal ideation who are diagnosed with prostate cancer should be offered additional psychological support.

## Introduction

Prostate cancer is the second most common cancer diagnosed in males internationally and the fifth leading cause of cancer death [1]. It is estimated that there are over 1.1 million incident cases of prostate cancer diagnosed annually worldwide [2]. The number of survivors globally is expected to increase rapidly into the future as the number of people aged 60 years or over is expected to double to 2 billion by 2050 [3].

Australia and New Zealand experience the highest rates of prostate cancer internationally with current data showing that 1 in 5 Australian men can expect a diagnosis to age 85 and 1 in 30 are expected to die from prostate cancer before age 85 [4]. The gap between the incidence and mortality rates has continued to grow since the introduction of the prostate specific antigen (PSA) test in the late 1980s [5]. While this has resulted in a much greater number of men surviving long after diagnosis [6], the population-wide psychosocial impacts of a much larger pool of men living with the consequences of cancer diagnosis and treatment is considerable. For example, between thirty and fifty percent of prostate cancer survivors report unmet needs for sexuality, psychological, health system and information [7, 8]. Long term prostate cancer survivors report high levels of psychological impacts that can include distress, worry, pain, dyspnoea, appetite loss, constipation, diarrhea and financial difficulties [9]. Medium to long-term quality of life and psychological well-being are associated with age, socio-economic status and marital status [10–12]. Several international studies have shown an increased risk of suicide after a diagnosis of prostate cancer [11–13] and after a diagnosis of lung, pancreatic, bladder and head and neck cancers [14].

The association between prostate cancer and suicide in Australia is not well documented. In 2015 suicide accounted for about 2.5% of total deaths for all Australian males [15]. Between 1997 and 2007 the average annual suicide rate for Australian males declined by 3.6% per year [16]. While death from suicide is relatively more common for younger men, the highest age-specific suicide death rate for Australian males was observed for those aged 85 years and over (39.3 per 100,000)[15].

We undertook a population-wide descriptive study using linked data to examine the relationship between prostate cancer and subsequent suicide. The primary aim of this study was to determine whether Australian men resident in New South Wales (NSW) and diagnosed with prostate cancer are at higher risk of suicide than men of the same age in the general population. The secondary aim was to determine whether disease or patient related characteristics are associated with suicide risk after prostate cancer.

## Materials and methods

### Participants

**Fig 1:**
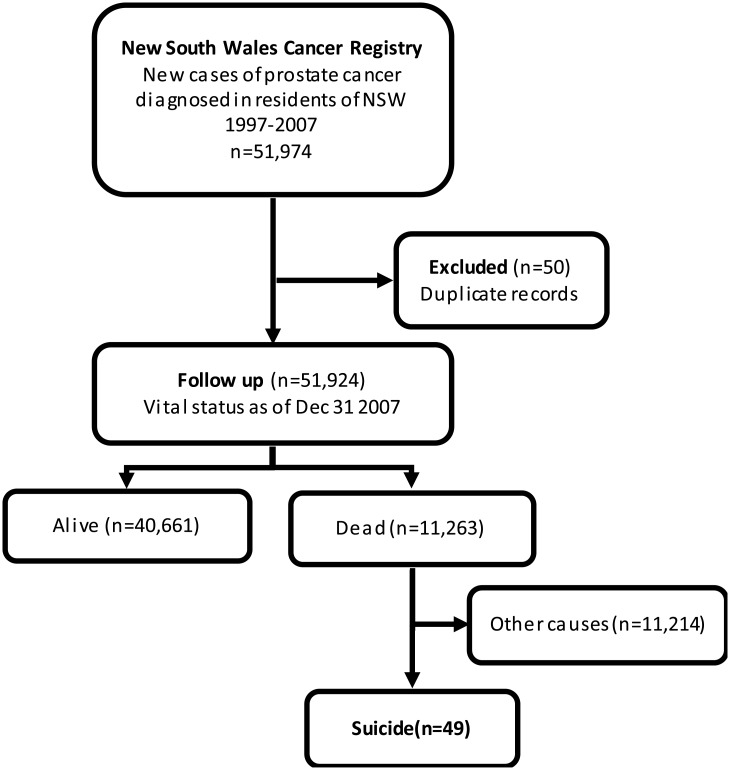
Flow chart for selection of study cohort.

Data for men diagnosed with prostate cancer (ICD-9 C61) between January 1997 and December 2007 were obtained from the NSW Cancer Registry (Fig 1). 2007 was the most recent year for which linked data were available. Notifications to the NSW Cancer Registry of all invasive prostate cancers (ICD-10 code: C61) diagnosed in NSW are mandated from pathology laboratories, hospitals, radiotherapy and medical oncology departments, aged care facilities and the Registry of Births, Deaths and Marriages under the Public Health Act 2010. NSW, the most populous state of Australia (approximately 7 million people in 2007) makes up one third of the Australian population. Information requested included month and year of diagnosis, date of birth, country of birth, place of residence and spread of disease at diagnosis. Spread of disease was classified as localised, or non-localised (a grouping of regional spread and distant metastases) or unknown according to the system described by Jensen et al. [17]. Cases diagnosed at or after death were excluded from this study.

### Follow up

Linkage between cancer notifications and deaths was undertaken. Deaths in the cohort were determined by linkage with records from the NSW Registry of Births, Deaths and Marriages and the National Death Index. We used the International Classification of Diseases version 9 (1997–1998) and 10 (1999–2007) to classify death from suicide (ICD 9 codes E950-E959 and ICD 10 codes X60-X84). Person-years at risk were calculated for the cohort of prostate cancer cases with the time-at-risk starting at diagnosis and ending at either death from suicide or from other causes, or the end of 2007, whichever occurred first.

### Record linkage

Linkage was also undertaken between cancer records and hospital admission data held by the Ministry of Health (Admitted Patient Data Collection). Data on marital status was obtained from these data sets. This linkage was performed by the Centre for Health Record Linkage (CHeReL) using name, address and date of birth and probabilistic matching carried out with ChoiceMaker software (Choice- Maker Technologies Inc., New York, US). Both certain and uncertain matches were reviewed clerically by CHeReL linkage officers, resulting in approximately 0.1% false positive and less than 0.1% false negative linkages.

### Data analysis

We constructed five-year age group by single calendar-year contingency tables for the men in the study cohort. Person-time at risk was calculated by aggregation of the survival times of men within each age group and calendar-year combination. The expected number of suicides in the study cohort was calculated by multiplying the age-, calendar-year- specific person-times at risk with the corresponding, age, sex and calendar period suicide rates in the entire NSW male population obtained directly from the Australian Bureau of Statistics (ABS). Standardised Mortality Ratios (SMRs) were calculated as the number of observed suicides divided by the expected number of suicides and 95% confidence intervals were calculated assuming the observed number of suicides followed a Poisson distribution [18].

Area level socio-economic status for each individual was derived from ABS census data on socio-economic disadvantage for the local government area of residence at the time of prostate cancer diagnosis [19]. Local government area at the time of diagnosis was also used to determine an accessibility or remoteness score for each man’s place of residence [ARIA+] [20].

Piecewise negative binomial regression analysis was used to estimate adjusted rate ratios (RR) of death from suicide among prostate cancer patients. The natural logarithm of the person years at risk of suicide was used as the offset. Variables that were found to be significantly associated with excess risk of death in the bivariable analysis were retained in the multivariable model. 95% confidence intervals for RRs were also calculated. Stage of diagnosis was grouped into localised, non-localised and unknown because only two men had distant spread).

All analyses were performed in SAS version 9.3 (SAS Institute Inc., Cary, NC, US). The NSW Population and Health Services Research Ethics Committee approved this study (Approval number HREC/11/CIPHS/49). Data are available through application to the NSW Cancer Registry and the Centre for Health Record Linkage.

## Results

There were 51,924 men diagnosed with prostate cancer in NSW between January 1997 and December 2007 (Table 1). These men contributed 196,146 person years of follow-up time during the study period. In total we observed 11,263 deaths from all causes up to the end of the follow-up period (December 2007). Forty-nine were classified as suicide, equating to a crude incidence rate of 25 suicides per 100,000 person years and an SMR of 1.70 (95% CI 1.26–2.25, Table 2).

**Table 1:** Demographic characteristics of New South Wales men diagnosed with prostate cancer in 1997 to 2007, comparing those who committed suicide with all men diagnosed with prostate cancer, number, percent, person years at risk and crude rate per 100,000 person years at risk.

	All men with prostate cancer N = 51,924		Deaths due to suicide N = 49		Person years at risk	Crude rate per 100,000	p-value*
	n	% of N	n	% of N			
**Age at diagnosis**							
**Median age at diagnosis**	**73 yrs**						
<65	15,872	30.6	12	24.5	63,774	18.8	0.064
65–74	19,516	37.6	17	34.7	81,497	20.9	
75 and over	16,536	31.8	20	40.8	50,875	39.3	
**Period of cancer diagnosis**							
1997–2000	14,671	28.3	21	42.9	94,956	22.1	0.698
2001–2004	18,377	35.4	20	40.8	75,029	26.7	
2005–2007	18,876	36.4	8	16.3	26,160	30.6	
**Years after diagnosis** ^							
0–1	51,924	100.0	26	53.1	46,513	55.9	<0.001
1–2	41,732	80.4	6	12.2	37,655	15.9	
2–4	33,576	64.7	9	18.4	53,570	16.8	
4+	20,618	39.7	8	16.3	58,407	13.7	
**Country of birth**							
Australia	33,475	64.5	30	61.2	124,711	24.1	0.732
Outside Australia	18,449	35.5	19	38.8	71,434	26.6	
**Accessibility of residence**							
Major city	34,283	66.0	40	81.6	132,172	30.3	0.038
Outside major city	17,641	34.0	9	18.4	63,973	14.1	
**Socioeconomic status**							
Most advantaged	17,197	33.1	14	28.6	67,327	20.8	0.450
Middle group	18,597	35.8	16	38.8	68,756	23.3	
Most disadvantaged	16,130	31.1	19	32.7	60,063	31.6	
**Stage at diagnosis**							
Localised	23,795	45.8	17	34.7	90,548	18.8	0.041
Non-localised	5,127	9.9	8	35.7	14,387	55.6	
Unknown	23,002	44.3	24	37.7	91,211	26.3	
**Marital Status**							
Married or living as married	37,347	71.9	20	40.8	143,016	14.0	<0.001
Single, divorced, widowed, separated	12,045	23.2	27	41.8	41,495	65.1	
Unknown	2,532	4.9	2	42.8	11,635	17.2	

*P-value from unadjusted piecewise negative binomial regression

^ Frequencies for categories of “years after diagnosis” sum to more than 51,924 because men can be at risk for multiple years after diagnosis.

**Table 2:** Observed and expected numbers of suicide deaths, Standardised Mortality Ratios (SMR) and 95% confidence intervals (95% CI) for New South Wales men diagnosed with prostate cancer during 1997 to 2007.

Group	Observed	Expected	SMR	95% CI
**Stage at diagnosis**				
Localised	17	13.60	1.25	0.73–2.00
Non-localised	8	2.22	3.61	1.56–7.11
Unknown	24	13.03	1.84	1.18–2.74
**Years after diagnosis**				
0	26	0.68	38.40	25.08–56.27
1	6	1.75	3.43	1.26–7.46
2–3	9	5.39	1.67	0.76–3.17
4 and over	8	21.03	0.38	0.16–0.75
**Total**	49	28.85	1.70	1.26–2.25

During the study period the median age at prostate cancer diagnosis was 73. The median age at death from suicide in this group was 74, compared to a median age at death of 80 for all other men with prostate cancer who died of any cause.

Eleven suicide deaths (22% of all suicides in this patient group) occurred within one month of diagnosis, 18 (37%) within three months and 26 (53%) occurred within one year of diagnosis.

**Fig 2:**
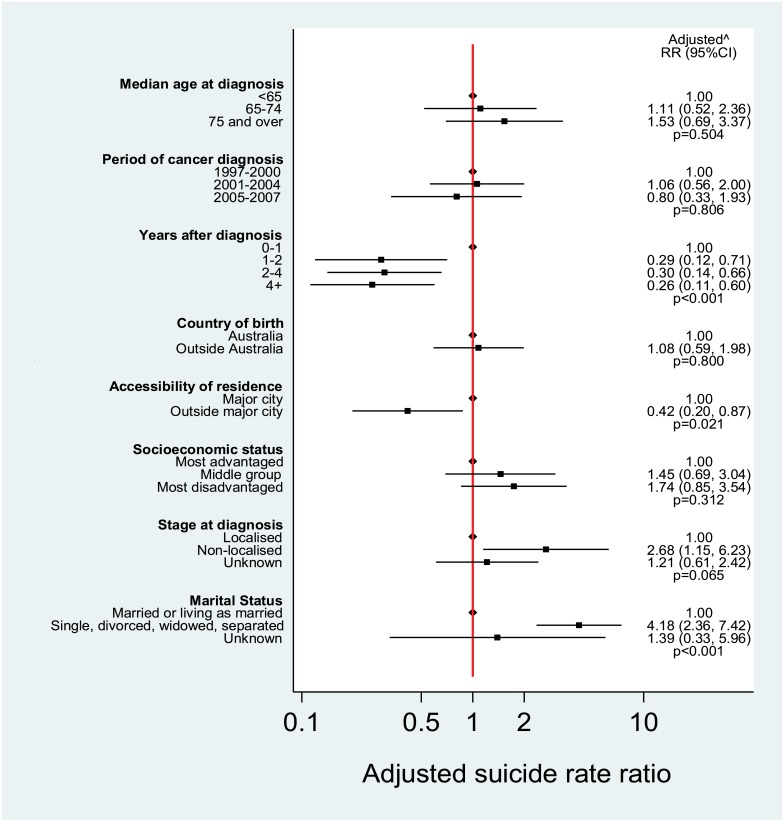
Forest plot of adjusted rate ratios (RRs) for suicide death (with 95% confidence intervals) for NSW men diagnosed with prostate cancer, 1997–2007. ^ Adjusted for median age at diagnosis, period of cancer diagnosis, years after diagnosis, country of birth, accessibility of residence, socioeconomic status, stage at diagnosis, marital status.

Number of years after diagnosis of prostate cancer, area of residence at diagnosis, stage at diagnosis and marital status were all associated with death from suicide in unadjusted analyses (p<0.001, p = 0.038, p = 0.041 and p<0.001 respectively)(Table 1). After adjusting for patient characteristics, risk of suicide diminished over time since diagnosis (RR in 1–2 years after diagnosis = 0.29, 95% CI: 0.12–0.71, 2–4 years RR = 0.30, 95% CI: 0.14–0.16 and 4+ years RR = 0.26, 95% CI: 0.11–0.60 compared with <1 year since diagnosis), men living outside major cities had lower risk of suicide compared to those resident in major cities (rate ratio = 0.42, 95% CI: 0.20–0.87) Men with non-localised disease had a higher risk of suicide compared to men with localised disease (rate ratio = 2.68, 95% CI: 1.15–6.23). Single, divorced, widowed or separated men were more likely to commit suicide than married men (RR = 4.18, 95% CI: 2.36–7.42) (Fig 2).

Most men (n = 27, 55%) who committed suicide were unmarried, divorced, separated or widowed. Of the group who were unmarried and committed suicide over 48% (n = 13) were widowed.

## Discussion

In this population-based cohort study of NSW men, we observed a 70% higher risk of suicide death for men diagnosed with prostate cancer compared to the general population. This risk was higher for men with non-localised disease (compared to localised), higher within the first year after diagnosis, in those who were unmarried and/or single men and men resident in major cities. The reasons for lower risk of suicide for men resident outside major cities is not clear but may be related to higher social isolation for men with prostate cancer in urban areas. We found no association between risk of suicide for men with prostate cancer and age, country of birth, or area-level socio-economic status. Our findings generally corroborate previous results from population-wide studies in Sweden and the USA [13, 21] that reported an elevated risk of death from suicide within the first year after diagnosis compared with cancer-free controls. Fall *et al*.[22] reported that the risk of suicide was in fact more than double that of cancer-free men (Relative risk = 2.6, 95% CI: 2.1–3.0). Notably, this risk was highest during the first week after diagnosis (RR = 8.4, 95% CI: 1.9–22.7) (20). A further study from the USA reported a higher risk of suicide for men with prostate cancer, when compared to men with other solid malignancies [12]. Prostate cancer has a relatively good prognosis compared with many other cancers, with five- year relative survival rates in excess of 95% in most developed countries. The increased risk of death from suicide in this large and growing population of men, particularly in the first twelve months after diagnosis implies that more could be done to communicate with patients and address the psychological well-being of vulnerable newly diagnosed men.

The psychological effect of a prostate cancer diagnosis is well documented. Men with prostate cancer may experience negative intrusive thoughts and other significant decrements to their psychological well-being [23]. Prostate cancer survivors have been shown to have an increased risk of depression [24, 25] and in other studies 12–18% of those newly diagnosed men reported suicidal ideation [26, 27]. While the factors that contribute to a completed suicide by cancer patients are complex, a broad understanding of predisposing factors can help those providing care for cancer patients to mitigate against this risk.

Our results indicated a higher risk of suicide for men with non-localised disease consistent with other studies. A Danish study of 564,508 cancer patients diagnosed between 1971 and 1986 identified that the highest risk of suicide was observed for patients with non-localised cancers and cancers with a perceived poor prognosis [28]. The higher risk of suicide for men with non-localised disease may be due to the perception that the hope of cure is out of reach, leading to a feeling of hopelessness [13]. Carlsson et al. reported that men with the highest risk disease had the highest risk of death from suicide in the first 6 months after diagnosis (Relative risk = 10), but this fell to 1.8 more than six months after diagnosis [11].

A possible contributing factor to risk of suicide is the administration of androgen deprivation therapy to men with locally advanced prostate cancer. A study of quality of life for asymptomatic men with non-metastatic prostate cancer found that androgen deprivation therapy resulted in increased fatigue, decreased physical activity, greater emotional distress, and poorer general health within the first year of commencing the treatment [29]. While the risk of suicide was increased for men receiving any treatment strategy compared to the general population, Carlsson reported the risk was particularly elevated for men treated with hormonal therapy (Relative risk = 6.0) and still elevated but less so for those treated with curative intent (RR 2.7) [11]. While we were unable to report on preexisting diseases the likelihood of clinically significant anxiety and depression has also been shown to be increased with the number of comorbid conditions, including pre-existing psychological conditions [30]. Up to 60% of suicides occur in people who suffer from depression [31].

Loneliness and social isolation have previously been shown to be associated with suicide in adult populations. We found that men diagnosed with prostate cancer who were unmarried, divorced, separated or widowed had a more than four-fold risk of suicide compared with their married or partnered peers. The risk of suicide for these single men appeared to be closer to the date of diagnosis than that for married or partnered men. This pattern may reflect a background trend in suicide risk that has been shown to be gender specific. A study based on the National Longitudinal Mortality study in the USA found that marital status, especially divorce, was a strong risk factor for mortality from suicide, but only for men [32]. This risk has been corroborated in an Australian study, where it was found that the sense of belonging gained by marital status was affected by gender and for older men in particular, widowhood was associated with lower levels of belongingness [33]. In the context of a significant life stressor, such as prostate cancer diagnosis, this emphasizes the importance of domestic supportive care and if this is not available, then other forms of psychological monitoring and care.

There are several limitations in the study. The number of suicides was small as the time period of the study was limited by the availability of complete data on the cause of death from the linked ABS file. A number of suicides will be underreported or classified as accidental deaths or death from other causes. Australian Bureau of Statistics data were estimated to underreport suicide deaths by between 11 and 16% in 2004 [34]. This is unlikely to be systematically different between those men with prostate cancer and those in the general population and therefore unlikely to bias the SMRs. Data on preexisting psychological conditions were not available. These data would have been particularly useful to examine whether prostate cancer patients with a prior history of anxiety, depression or suicidal ideation were at higher risk and if so by how much.

A higher risk of suicide has been observed in a number of patient groups including those with very poor prognosis cancers such as lung and pancreatic cancers and those with more favorable survival including bladder, head and neck and prostate cancer. Across this spectrum various clinical factors have been associated with suicidal ideation including depression, pain, insomnia, fatigue, loss of autonomy and independence, poor social support, impaired physical functioning, demoralization, and emotional distress [35].

Although we identified a relatively small number of cases, the community-wide impact of suicide is widespread. Suicide is only one manifestation of the impact of the psychological consequences of prostate cancer. Counting completed suicides fails to enumerate the full spectrum of psychological consequences for these men, however it does define the most serious consequence that can occur. Doctors and health care providers need to be mindful of the psychosocial context in which cancer is diagnosed and flag at-risk, ‘rescuable’ prostate cancer survivors for close monitoring [36]. This might involve clinicians working closely with mental health professionals, social workers and nurses to identify and address the unique and personal impact prostate cancer has on men’s lives [37].

How potentially vulnerable men are identified and provided with a practical toolkit of measures to reduce this risk is of high priority. Identification of men at risk as early as possible in the diagnostic pathway to ensure assessment and appropriate referral to psychosocial care should be an integral part of the patient’s care plan [38] and indeed has been accepted as a standard of care internationally [39]. The use of a brief, simple, no cost, Distress Thermometer has been recommended with a prostate cancer specific version well validated for men with prostate cancer [40] and available from the Prostate Cancer Foundation of Australia. (http://www.prostate.org.au/media/458256/Prostate_Cancer_Distress_Form.pdf) Additionally, a new screening algorithm using measures of self-reported quality of life, erectile dysfunction and depression has been proposed to assist in screening patients at high risk of suicide after prostate cancer diagnosis.[41]

If anxiety, distress or depression are suspected or identified, a number of interventions can be activated, by any member of the health care team, depending on the man’s needs. These can include referral to appropriate care, peer support, exercise programs or group-based interventions [37].

## Conclusion

Although the absolute risks of suicide for Australian men with prostate cancer are modest, in relative terms the 70 percent increased risk in this group of men is of concern. Our findings reflect only the tip of the iceberg in the spectrum of psychological stress that men with prostate cancer experience after diagnosis and indicate that those men who appear particularly vulnerable and who lack social support would benefit from interventions to prevent suicide after a diagnosis of prostate cancer.
